# Chitinase-like Proteins YKL-40 and YKL-39 in Colorectal Cancer

**DOI:** 10.3390/cells15030263

**Published:** 2026-01-30

**Authors:** Tsvetomira Ivanova, Maria Kazakova, Dorian Dikov, Angel M. Dzhambov, Nikolay Belev, Boyko Atanasov, Victoria Sarafian

**Affiliations:** 1 Department of Medical Biology, Medical University-Plovdiv, 4002 Plovdiv, Bulgaria; victoria.sarafian@mu-plovdiv.bg; 2 Research Institute at Medical University-Plovdiv, 4000 Plovdiv, Bulgaria; boyko.atanasov@mu-plovdiv.bg; 3 Department of Pathology, Jossigny Hospital, 75000 Jossigny, France; ddikov@ghef.fr; 4 Environmental Health Division, Research Institute at Medical University-Plovdiv, 4002 Plovdiv, Bulgaria; angel.dzhambov@mu-plovdiv.bg; 5 Department of Propaedeutics of Surgical Diseases, Medical University-Plovdiv, University Hospital Eurohospital, 4002 Plovdiv, Bulgaria; nikbel.vm@gmail.com; 6 Department of Propaedeutics of Surgical Diseases, Medical University-Plovdiv, Unihospital, 4002 Panagyurishte, Bulgaria

**Keywords:** YKL-39, YKL-40, CRC, tumor budding, liquid biopsy

## Abstract

YKL-40 and YKL-39 chitinase-like proteins (CLPs) are secreted glycoproteins involved in inflammation, macrophage polarization, and carcinogenesis. Their expression is significantly upregulated in various inflammatory and immunological conditions, including several cancers, suggesting a role as potential diagnostic markers. Colorectal cancer (CRC) remains a significant global health concern, with a continued need for reliable biomarkers to stratify patients and predict therapy response. In this study, we assessed tissue, plasma, and transcript levels of both CLPs in CRC. We found a strong association between their tissue expression and tumor budding. Notably, plasma YKL-39 levels were lower in CRC patients than in controls, while YKL-40 concentrations were higher in the patient group. Gene expression analysis for both CLPs in white blood cells (WBCs) did not reveal statistical significance between CRC patients and controls. These findings enhance our understanding of the clinical relevance of these molecular signatures and support their potential application as biomarkers in CRC stratification.

## 1. Introduction

Colorectal cancer (CRC) is the second leading cause of cancer deaths worldwide. By 2040, the number of CRC cases is predicted to reach 3.2 million [1]. The histopathological examination is still considered the most essential approach for diagnosis, clinical management, and oncology research.

Reliable prognostic biomarkers to better stratify patients and predictive biomarkers of treatment response are currently lacking. The chitinase-like proteins (CLPs) were found to be involved in various processes related to tissue remodeling, angiogenesis, and inflammation [2,3,4]. The CLPs are evolutionarily preserved molecules with diverse functions, although their enzymatic activity has been lost in mammals [5].

YKL-40, also known as Chitinase-3-Like Protein 1 (CHI3L1), is a highly conserved glycoprotein [6]. It is used as a marker for the differentiation and activation of macrophages and microglia [7]. The protein is usually expressed by many cell types, including macrophages, chondrocytes, and vascular smooth muscle cells [2,8]. Its role in promoting angiogenesis and in the development of various neoplasms, such as breast cancer, CRC, and glioblastoma, is already established [9,10]. In our previous study, we have shown that increased YKL-40 expression in glioma is associated with tumor aggressiveness and stage [11,12].

Another member of this protein family, YKL-39 (Chitinase-3-Like Protein 2 (CHI3L2) [13], participates in the regulation of autoimmunity and tissue remodeling [14]. Studies have shown that its concentrations are elevated in degenerative pathologies, including osteoarthritis, multiple sclerosis, Alzheimer’s disease, and amyotrophic lateral sclerosis [15,16,17]. YKL-39 exhibits monocyte chemotactic and pro-angiogenic activity. Altered YKL-39 levels have also been observed in several cancers, including breast, glioma, and kidney cancers, where elevated amount is associated with poor prognosis and implication to tumor angiogenesis [18,19,20]. There are still no accessible reports for the significance of YKL-39 in CRC.

Secreted YKL-39 and YKL-40 have been proposed as potential prognostic biomarkers associated with progression and recurrence of some neoplasms [21]. It was shown that their variable expression is influenced by cancer type, disease stage, and intratumoral heterogeneity [22]. The budding phenomenon, another biological characteristic of tumor cells, has not been investigated in the context of CLPs yet. It is observed in tumorous tissues in several malignancies and is defined as the presence of single up to four cancer cells located at the invasive front of the tumor [23]. It was proposed that tumor buds illustrate the epithelial–mesenchymal transition (EMT) linked to tumor aggressiveness [23]. Tumor buds are suggested as a poor prognostic marker for CRC, but they have not been incorporated into routine clinical practice yet. Furthermore, no data is currently available regarding the correlation between YKL-40 and YKL-39 expression in CRC and their connection with tumor budding.

The current pilot study evaluates CLP protein and gene expression alongside tumor budding and presents new evidence of their association in CRC.

## 2. Materials and Methods

### 2.1. Materials

#### Patients and Samples

Samples from clinically and histologically diagnosed CRC patients were collected from the Department of General and Clinical Pathology, Medical University—Plovdiv, Bulgaria (*n* = 32), during the period of January 2022 to December 2024. Medical records were utilized to gather demographic data (age, sex) and detailed clinicopathological information, including tumor localization, histological grade, pTNM tumor stage, lymphatic and vascular invasion, mutation status, microsatellite instability (MSI), and tumor budding. Notably, none of the patients had undergone prior radiotherapy or neoadjuvant therapy. Tissue samples from CRC patients were examined alongside control samples. The internal control consisted of normal colonic tissues obtained from areas distal to the tumor site in CRC patients. Resected colonic tissue was fixed in 10% neutral buffered formaldehyde and embedded in paraffin. Four 4 µm thick paraffin sections were prepared for immunohistochemical analysis and hematoxylin-eosin (HE) staining. Two pathologists (DD and VS) independently assessed tumor budding, blinded to patient outcomes, in accordance with the tumor budding scoring guidelines of the 8th edition of the American Joint Committee on Cancer guidelines and International Tumor Budding Consensus Conference. Cases positive for tumor budding were categorized into three groups: Bd1 (0–4 buds/0.785 mm^2^), Bd2 (5–9 buds/0.785 mm^2^), and Bd3 (≥10 buds/0.785 mm^2^) [24]. For each patient, ten independent high-power fields were evaluated at ×400 magnification in the hotspot areas of the invasive tumor front. In cases of discordance, the slides were reviewed to achieve agreement. Quantitative interobserver agreement metrics were not applied in the present study, as over the past two decades, the assessment of the tumor budding in CRC has become a routine component of daily diagnostic practice among conventional pathologists. Furthermore, data from the current literature demonstrate an almost perfect intra-observer agreement following consensus application of standardized criteria [25]. In rare cases where histological consensus could not be reached, immunohistochemistry (IHC) with cytokeratin 20+/cytokeratin 7-/CDX2+ markers was employed, in accordance with current guidelines [26].

The study received approval from the Ethics Committee of the Medical University of Plovdiv (Protocol No. 4/from 8 June 2022). Signed informed consent was obtained from all participants.

### 2.2. Methods

#### 2.2.1. Immunohistochemical Evaluation of YKL-40 and YKL-39

IHC was performed using the Novocastra^TM^ Peroxidase Detection System (Leica Biosystems, Deer Park, IL, USA, Cat. No. RE7110-K). After antigen retrieval, endogenous peroxidase was blocked, followed by protein blockage. All samples were incubated with primary anti-human YKL-40 and YKL-39 antibodies at 4 °C overnight. The rabbit polyclonal anti-YKL-40 antibody (Abcam, Cambridge, UK, ab180569) was applied at working dilution 1:100, and the rabbit recombinant anti-YKL-39 antibody (Assay Genie, Dublin, Ireland, PACO43992) at 1:200. Tissue samples were then treated with biotinylated secondary antibodies and DAB chromogen as previously described [12]. The primary antibodies repeatedly demonstrate the expected localization across all positive samples [27]. Omission of the primary antibody was used as a negative control.

IHC staining was initially evaluated using a light microscope with a ×10 objective (magnification ×100) to scan and identify histologically the hotspot tumor areas within the tumor parenchyma (central tumor zone) and the invasive front exhibiting tumor budding. Subsequently, a ×40 objective (magnification ×400) was used for detailed assessment of staining. Relative immunohistochemical protein expression levels were determined semi-quantitatively using the histochemical score (h-score) assessment, as described in and modified from previously published literature immunohistochemical scoring methods [28,29].

Briefly, both staining intensity (0 = no evidence of staining, 1 = weak staining, 2 = moderate staining, and 3 = strong staining) and the percentage of stained cells (from 0 to 100%) at each intensity level were evaluated. The score was obtained by the formula: 3 + (3 × percentage of strongly stained cells); 2 + (2 × percentage of moderately stained cells), and 1 + (1 × percentage of weakly stained cells), with a range of h-score of 0 to 300 (1, 2). A marker was considered positive when its h-score was ≥10. Expression with low grade h-score was considered from 10 to 100; moderate, from 100 to 200, and high, from 200 to 300. In our statistical analysis of the results, we evaluated expression using abbreviated indices 1 (low), 2 (moderate), and 3 (high). The concordance of continuous variables was assessed using the average intraclass correlation coefficient (ICC) based on a two-way random-effects model. According to the 95% confidence interval of the ICC estimate, values less than 0.5 indicate poor reliability, values between 0.5 and 0.75 indicate moderate reliability, values between 0.75 and 0.9 indicate good reliability, and values above 0.90 indicate excellent reliability [30].

#### 2.2.2. Isolation of Plasma and White Blood Cells (WBCs)

Peripheral blood was collected from the CRC patients before surgery, as well as from healthy controls. Plasma was isolated, aliquoted, and stored at −80 °C before analysis. WBCs were isolated by cold lysis of erythrocytes using a lysing solution with ammonium carbonate, ammonium chloride, and EDTA, according to the manufacturer’s instructions. The WBC pellet was resuspended in PBS, centrifuged for 10 min at 1800 rpm at 4 °C, and preserved at −80 °C for gene expression analysis.

The control group for blood samples consisted of 32 age- and sex-matched healthy volunteers (with a mean age of 48.9 ± 48.9 ± 14.4, of which 56.7% were females), with no history or current clinical evidence of disease. In accordance with the exclusion criteria, individuals with acute or chronic infections, inflammatory conditions, autoimmune disorders, or a history of malignancy were excluded. Comprehensive demographic data and medical histories were documented for all participants.

#### 2.2.3. RNA Gene Expression Analysis

Total RNA was isolated from WBCs using TRIzol Reagent (Thermo Fisher Scientific, Waltham, MA, USA, Lot. No. 1559602). Chloroform was added to the TRIzol-homogenized cells, and after centrifugation, the aqueous phase containing total RNA was transferred to a new tube. RNA was precipitated with isopropanol, washed with 75% ethanol, and solubilized in DNase/RNase-free water. To eliminate residual DNA, samples were treated with the TURBO DNA-free kit (Thermo Fisher Scientific, Waltham, MA, USA, Lot. No. AM1907). RNA was mixed with 3 μL TURBO DNase Buffer and 1.5 μL TURBO DNase Enzyme, incubated for 50 min at 37 °C, and treated with DNase Inactivation Reagent. Following centrifugation, the supernatant containing RNA was collected. RNA concentrations were determined spectrophotometrically (A260/280 ratio) using a NanoDrop 2000 (Thermo Fisher Scientific, USA). Gene expression in WBCs was assessed using the RevertAid^TM^ First-strand cDNA Synthesis Kit (Thermo Fisher Scientific, USA, Cat. No. K1632) to synthesize cDNA from 1 μg RNA. Total RNA was reverse transcribed using random Oligo(dT) primers. RT-PCR was performed using a three-step incubation protocol: 10 min at 42 °C, 45 min at 50 °C, and 10 min at 70 °C for heat inactivation of the reverse transcriptase enzyme. Samples were cooled to 4 °C, and cDNA was stored at −20 °C. The qPCR assay was designed in accordance with the Minimum Information for Publication of Quantitative Real-Time PCR Experiments (MIQE) guidelines [31]. qRT-PCR analysis of CHI3L1 and CHI3L2 (using 6.25 ng cDNA) was conducted with Green MasterMix (Genaxxon bioscience GmbH, Ulm, Germany, Cat. No. M3023.0500) according to the manufacturer’s instructions. Each sample was analyzed in duplicate using a Rotor-Gene Q 600 real-time PCR detection system (Qiagen, Hilden, Germany). Given the high metabolic turnover inCRC) and to prevent confounding effects from tumor growth on YKL gene expression, data were normalized to GAPDH, ACTINB, and UBC, which served as endogenous controls. Primer sequences for all genes are provided in Supplementary Table S1. All primer pairs demonstrated efficiency within the 90–110% range. Relative gene expression was calculated using the comparative 2^−ΔΔCt^ method and normalized to GAPDH, ACTINB, and UBC gene levels. Upon completion, a melting curve was generated by cooling to 35 °C at 20 °C/s, holding at 35 °C for 30 s, and then heating at 0.2 °C/s until 85 °C.

#### 2.2.4. ELISA for Detection of Plasma Levels of YKL-40 and YKL-39

Plasma protein levels of YKL-40 (Elabscience Human GP39 YKL-40, Cat. No. E-EL-H0037 Lot. No. CV0, Houston, TX, USA, with a detection range of 5.4–300 ng/mL) and YKL-39 (Circulex Human YKL-39 Cat. No. CY-8087, Woods Hole, MA, USA, with a detection range of 37.5–2400 pg/mL) of both CRC patients and controls were measured by ELISA. The tests were performed in duplicate following the manufacturer’s instructions. The intra-assay coefficients of variation (CV) were 10%, and the inter-assay CVs were <12%. The assay employs the sandwich-based ELISA method with optical density measured at 450 nm on Sunrise ELISA Reader (Tecan, Mannedorf, Switzerland).

### 2.3. Statistical Analysis

Comparisons between qRT-PCR and ELISA-derived YKL levels between the CRC and control groups were conducted using Mood’s median non-parametric test, as the dependent variables were not normally distributed according to the Shapiro–Wilk W test. The median test, albeit conservative, was used because it imposes no assumptions on the data’s distribution or on the homogeneity of variance. In addition, these bivariate tests were supplemented with quantile regressions adjusting for the effect of patient group for age and sex. Quantile regressions also make no distributional assumptions, but unlike the median test, allow for modeling the effect on the median of the dependent variable, controlling for confounding variables. The data was presented as the median with percentiles. Statistical data processing was conducted utilizing the Fisher–Freeman–Halton exact test to compare the differences in the protein expression in distinct tissue sections. Given that most variables were binary or ordered categorically, we used Kendall’s rank correlation analysis to explore the associations between YKL-40 and YKL-39 plasma protein, gene transcript levels in WBCs, tissue expression, and other clinical variables in patients with CRC. This approach assumed that the observed ordinal variables represent continuous latent constructs. Partial Kendall’s Tau-b correlations were computed by partialling out the variance explained by patients’ age and sex. In addition to asymptotic *p*-values, we calculated bias-corrected and accelerated bootstrapping 95% CI for these correlations based on 1000 replicates. In all statistical evaluations, associations were deemed statistically significant at the *p* < 0.05 level (two-tailed) or when the 95% CI excluded zero. Family-wise Benjamini–Hochberg correction was made for *p*-values within each analysis block (Fisher-Freeman–Halton tests, median tests, quantile regressions, and correlation tests). As IHC was performed on 20 CRC patient samples, pairwise deletion was used, and the analytical sample size varies across analyses. The analyses were conducted using Stata MP version 18, R version 4.5.2, and GraphPad Prism 10.

## 3. Results

### 3.1. Clinicopathological Characteristics of CRC Patients

Clinical data obtained from medical records play an important role in advancing our understanding of CRC disease progression and potential biomarkers. The mean age of CRC patients studied was 71.2 years. Of that, 53.1% were female. The clinicopathological characteristics of the patients are summarized in Table 1. Over 90% of the CRCs were moderately differentiated G2, where 87.5% were in pT3. Additionally, above 40% had pN1 and pN2 lymph node invasion. Currently, the observed survival time following the initial diagnosis ranged from 3 to 12 years.

Tumor budding was classified as high (Bd2 and Bd3) in 18 of 20 patients (90%). Absence of tumor buds (Bd0) was observed in 1 of 20 cases (5%), while low tumor budding was identified in 1 patient (5%). Among the 18 patients with high tumor budding, 11 (61.1%) showed no lymph node involvement (pN0), whereas the remaining 38.8% were classified as pN1 and pN2 (Supplementary Figure S1). The interobserver concordance study demonstrated a high level of agreement (≥90%) in the assessment of tumor budding, consistent with findings reported in the current literature [25].

### 3.2. Dysregulated YKL-40 and YKL-39 Protein and Gene Levels in Blood Samples of Patients with CRC

To assess the significance of the two CLPs in the pathogenesis of CRC, we evaluated, in parallel, both protein and gene expression levels in plasma samples and WBCs, respectively.

**Figure 1:**
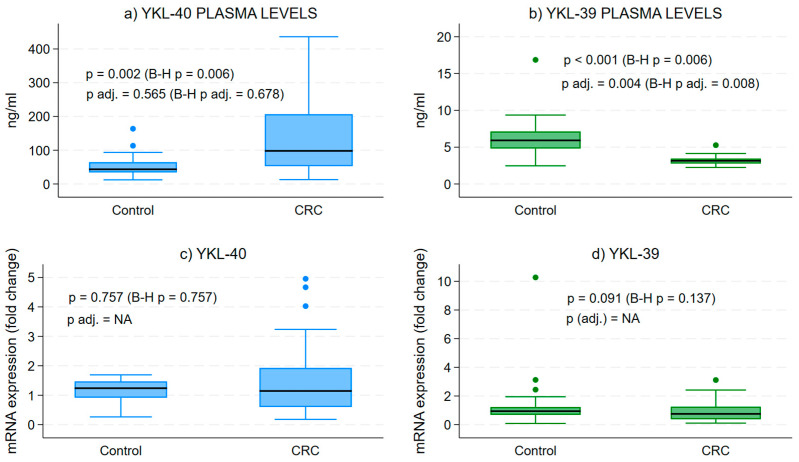
Protein and gene expression levels of YKL-40 and YKL-39 in blood samples. Difference in the median secretory plasma circulating forms of YKL-40 and YKL-39 in CRC and in healthy patients. Medians across groups are compared using Mood’s median test and quantile regressions, with the latter adjusting for age and sex. Analytical sample size: *N* = 64 in (panel **a**), from which *n* = 32 CRC and *n* = 32 control samples; *N* = 53 in (panel **b**), from which *n* = 29 CRC and *n* = 24 control samples; *N* = 52 in (panel **c**), from which *n* = 32 CRC and *n* = 20 control samples; and *N* = 59 in (panel **d**), from which *n* = 32 CRC and *n* = 27 control samples; p—*p* value from bivariate median test, p adj.—*p*-value from quantile regression, B-H p/p adj.—Benjamini–Hochberg corrected version of the median test or quantile regression-associated *p* value, NA—quantile regression effect estimates for CRC vs. control group could not be estimated due to collinearity in the models.

The examination of plasma protein levels for YKL-40 and YKL-39 demonstrated a substantial increase in YKL-40 concentrations when compared to the control group (Figure 1a). In contrast, we observed a notable decrease in the secretory form of YKL-39 in CRC patients relative to healthy individuals (Figure 1b).

Gene expression analysis in WBCs from CRC patients illustrated a broad range of transcript levels for both CLPs compared to controls (Figure 1c,d). However, the mean expression levels were lower than those of healthy individuals, though not significantly different.

### 3.3. YKL-40 and YKL-39 in CRC and Normal Tissues

In addition to the evaluation of protein and transcript levels in preoperative blood samples, we assessed the tissue and spatial expression of both CLPs in paraffin-embedded sections. The IHC analysis revealed significantly elevated expression in CRC tissues compared to normal colon mucosa samples. Notably, within the normal colon glandular parenchyma of CRC patients, there was an absence (0) or low (1) expression, with only rare instances of moderate (2) reactivity observed.

**Figure 2:**
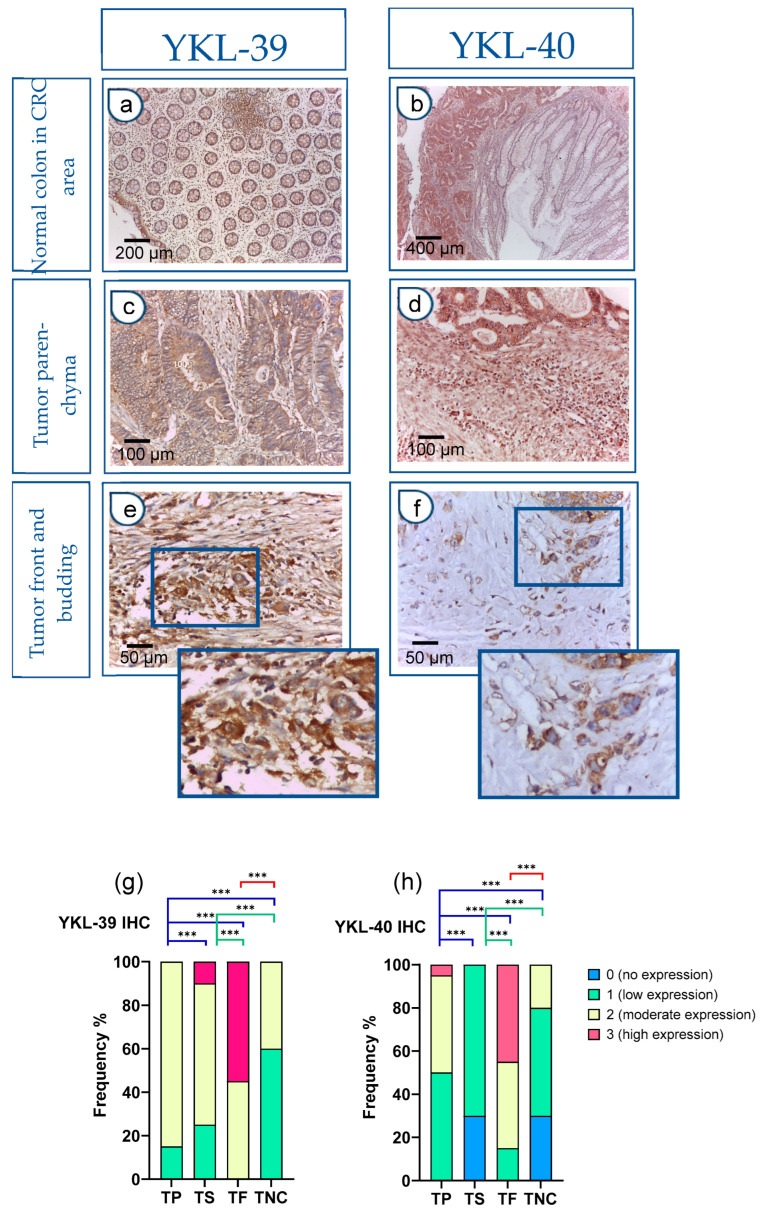
Strong YKL-40 and YKL-39 expression in tumor buds within the front of tumor invasion in CRC. (**a**–**f**) IHC on tissue sections. Normal colon tissue in the CRC area (**a**,**b**): (**a**) Normal colon mucosa: glandular epithelium 1+; stromal macrophages 2+. *IHC, YKL-39, ×100 (original magnification)*; glandular tumor parenchyma 3+ (left); absence of reactivity (0) in the mucosal chorion of normal colon (right); (**b**) Normal colon mucosa (right): glandular epithelium (0–1+), in contrast to tumor parenchyma (left) (3+). *IHC, YKL-39, ×50 (original magnification)*; Tumor parenchyma in the CRC tissue area (**c**,**d**): (**c**) Tumor parenchyma 3+, stroma 2+. *IHC, YKL-40, ×200 (original magnification)*; (**d**) Tumor parenchyma with clearly visible expression gradient 2+. *IHC, YKL-39, ×200 (original magnification)*; Tumor front and budding (**e**,**f**): (**e**) Front of tumor invasion 3+. *IHC, YKL-40, ×400 (original magnification)*; (**f**) Tumor front (Budd 3) with more pronounced expression in tumor cells. *IHC, YKL-39, ×400 (original magnification)*; YKL-40 and YKL-39 expression levels in tumor parenchyma, stroma, and front are assessed on adenocarcinomatous cells (cytokeratin 20+/cytokeratin 7-/CDX2+ markers). (**g**,**h**) Difference in the intensity of YKL-39 and YKL-40 expression in tumor parenchyma, stroma, and front in CRC and in normal colon. Statistically significant differences in the protein expression levels in the tumor parenchyma and in the tumor stroma relative to the tumor front and negative controls, respectively. Expression levels were categorized into four groups (category 0—no expression, 1—low expression, 2—moderate expression, 3—high expression) in the tissue regions analyzed: (TP—tumor parenchyma; TS—tumor stroma; TF—tumor front; TNC—normal colon tissue in the CRC area). Analytical sample size for YKL 40, *N* = 20; YKL-39, *N* = 20. *Fisher-Freeman-Halton test*. Differences are statistically significant with a Benjamini–Hochberg corrected *p*-value (*** *p* < 0.001). For exact uncorrected and corrected *p*-values, see Supplementary Table S2).

Figure 2a,b depicts the immunohistochemical staining, which demonstrates the presence of YKL-39 and YKL-40 in the normal colonic tissue distal to the tumor site of CRC patients. There was a low expression (1+) in the glandular epithelium and moderate expression (2+) in stromal macrophages. In contrast, the expression levels of both molecules were markedly elevated within the tumor parenchyma of CRC tissue samples (Figure 2c,d). Moreover, strong positive reactivity was prominently observed at the tumor front of invasion, particularly within tumor budding (Figure 2e,f).

The h-score was used to interpret YKL expression. In control patients, YKL expression was absent or showed a low h-score and a low ICC (0.35). Notably, in the main cohort of patients with CRC, the h-score was moderate for YKL-39 (ICC = 0.6) or high for YKL-40 (ICC = 0.9).

Next, we conducted a quantitative comparison of YKL-39 and YKL-40 expression levels across tumor regions, including tumor parenchyma, stroma, and the invasive front, in adenocarcinoma cells. Statistically significant differences were observed among all examined tumor and normal colon regions. Particularly intense staining was noted at the tumor invasion front when compared to the tumor parenchyma and noncancerous tissue (Figure 2g,h).

### 3.4. Correlation of YKL-40 and YKL-39 with Tumor Budding and Clinicopathological Markers

**Figure 3:**
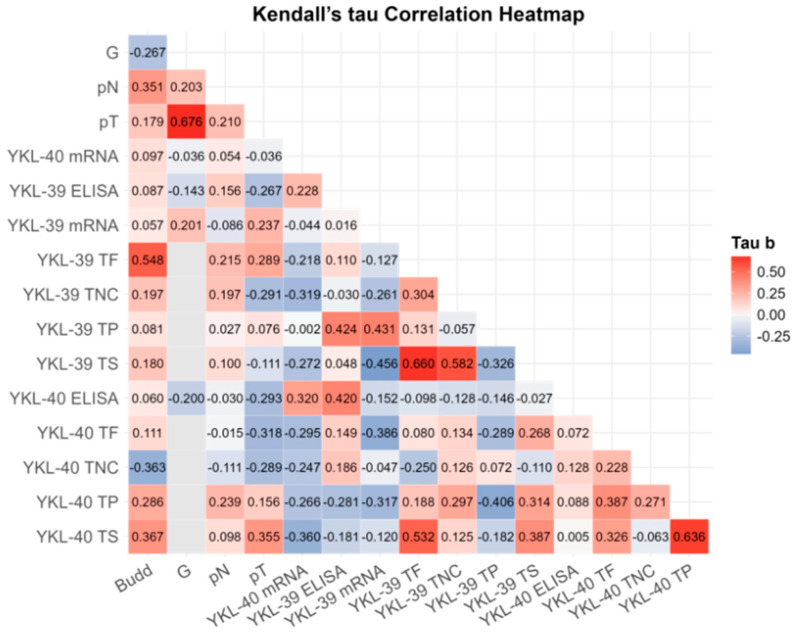
Kendall’s Tau rank partial correlation matrix between YKL-40 and YKL-39 plasma protein, gene transcript levels in WBCs, tissue expression, and other clinical variables in patients with CRC. Legend: G—grade of differentiation; pT—tumor stage; pN—invasion in lymph nodes; ELISA—plasma protein analysis; mRNA—gene expression; TP—tumor parenchyma; TS—tumor stroma; TF—tumor front; TNC—normal colon tissue in the CRC area. Analytical sample size: *N* = 32 for CRC YKL-40 plasma; *N* = 29 for CRC YKL-39 plasma; *N* = 32 for CRC YKL-40 mRNA; *N* = 32 for YKL-30 CRC mRNA; *N* = 32 for G, pN, pT; *N* = 20 for Budd, YKL-40 TP/TS/TF, YKL-39 TP/TS/TF. Correlation coefficients are adjusted for age and sex. For exact uncorrected and corrected *p*-values, see Supplementary Table S3).

To investigate the potential relationship between blood and tissue expression levels, tumor budding, and clinicopathological parameters, we conducted a correlation analysis (Figure 3). Following a correction for multiple comparisons, robust significant correlations were observed only between G and pT (r = 0.676; BCa 95% CI: 0.239, 0.756; *p*_B-H_ = 0.003), between YKL-40 in the tumor parenchyma and the tumor stroma (r = 0.636; BCa 95% CI: 0.351, 0.849; *p*_B-H_ = 0.014) and between YKL-39 in the tumor front and the tumor stroma (r = 0.660; BCa 95% CI: 0.392, 0.870; *p*_B-H_ = 0.012) (Supplementary Table S2). While tumor budding strongly correlated with YKL-39 expression at the tumor front (r = 0.548; BCa 95% CI: −0.112, 0.796; *p*_B-H_ = 0.048), it was also associated with YKL-40 expression in the tumor stroma (r = 0.367, *p* = 0.040) and lymph node involvement (r = 0.341, *p* = 0.058). These findings; however, turned out to be not significant after the Benjamini–Hochberg correction.

Positive associations were detected between YKL-39 and YKL-40 across various tumor regions. Notably, we identified correlations between YKL-39 in the tumor front and YKL-40 in the tumor stroma (r = 0.532, *p* = 0.004) and between both chitinases in the tumor stroma (r = 0.387, *p* = 0.030). However, these correlations did not remain significant after the B-H correction.

### 3.5. Discriminative Power of YKL-40 and YKL-39

**Figure 4:**
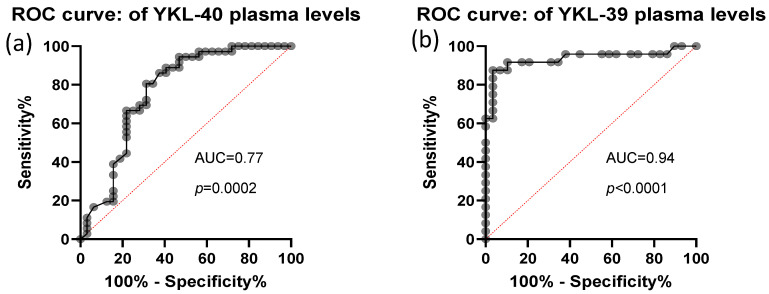
ROC analysis of CRC plasma YKL-40 and YKL-39 levels. Analytical sample size: *N* = 67 in (panel **a**), from which *n* = 32 CRC and *n* = 35 control samples; *N* = 53 in (panel **b**), from which *n* = 29 CRC and *n* = 24 control samples. Median and 25–75 percentile values of YKL-39 and YKL-40 are 3.68 ng/mL (3.08–5.58) and 54.05 ng/mL (35.94–100.11), respectively.

The predictive value of plasma YKL-40 and YKL-39 was assessed using ROC curve analysis. Both biomarkers demonstrated high discriminative power, with AUC values of 0.77, STDEV-0.06, *p* = 0.0002 for YKL-40 and AUC values of 0.94, STDEV-0.04, *p*< 0.0001 for YKL-39 (Figure 4). These findings indicate that plasma levels of both CLPs are strong candidates for distinguishing CRC patients from healthy individuals.

## 4. Discussion

Given the emerging role of liquid biopsy in cancer detection, as well as in molecular profiling and treatment response prediction, we evaluated the protein and gene expression levels of both YKL-40 and YKL-39 CLPs in blood samples. To our knowledge, this is the first study to report in parallel the transcriptional and protein levels of both CLPs in WBCs and plasma within the context of CRC. The gene expression analysis in WBCs from the CRC patients studied showed a wide range of transcript levels for both markers compared to controls, with mean expression lower than in healthy individuals, though not statistically significant. WBCs serve as valuable tools in biomedical research, offering insights into immune health, disease status, and therapeutic responses by revealing gene and protein expressions associated with immunity, inflammation, and cancer [32]. WBCs are particularly suitable for identifying early diagnostic or prognostic non-invasive biomarkers for various diseases. It is hypothesized that WBCs may reflect persistent inflammation in CRC, thereby indirectly influencing the tumor microenvironment, a view supported by other researchers [33]. In our study, the absence of significant differences in the RNA expression of YKL-39 and YKL-40 in the WBCs of CRC patients compared to healthy controls is noteworthy. The most plausible explanation for this finding is that the primary producers of these proteins are not the circulating blood cells, as previously indicated [2].

Interestingly, our analysis revealed a significant increase in YKL-40 plasma protein concentrations in CRC compared to the control group. Conversely, we observed a notable decrease in the secretory form of YKL-39 among CRC patients relative to healthy individuals. Lower YKL-39 levels have also been previously reported in conditions such as bronchial asthma [34]. Therefore, the regulatory mechanisms underlying the observed reduction in the half-life of circulating YKL-39 warrant further investigation and analysis.

In a previous study involving another CRC patient cohort, we observed strong YKL-40 expression in tumor cells, particularly at the tumor front, which itself was associated with lymph node involvement [12]. In the current CRC study group, we examined the spatial tissue expression patterns of YKL-40 and YKL-39. Both CLPs exhibited similar localization, with high levels of immunostaining detected in the tumor front. Of note, we identified high-grade tumor budding in the majority of the patients included in our study. This finding may explain the relatively high frequency of regional lymph node metastases observed. We found a strong association between CLPs’ tissue expression and tumor budding, which may likewise be attributed to the large proportion of cases with high-grade tumor budding (Bd2 and Bd3), a factor that undoubtedly influenced the statistical outcomes. On the other hand, this observation may be regarded as indirect evidence supporting the role of CLPs in CRC progression and metastasis at the invasive tumor front.

The significance of YKL-39 in CRC is still largely unknown. Several research groups highlight the role of this protein in addition to angiogenesis in supporting metastasis [18,35]. Conversely, evidence indicates that in breast tumor tissue, YKL-39 is primarily produced by macrophages and does not correlate with clinicopathological markers or patient survival [36]. Despite this fact, YKL-39 expression decreases with higher tumor grade and with negative estrogen (ER) and progesterone receptor (PR) status. These findings contrast with those of Liu et al. and Kzhyshkowska et al., who report that YKL-39 serves as an unfavorable prognostic factor in cancer cells [18,35]. Furthermore, YKL-39 has been proposed as a biomarker for chondrocyte activation and osteoarthritis progression [37,38].

On the other hand, YKL-40 is among the most extensively studied CLPs and is recognized for its pro-angiogenic effects in various cancers, including colon and breast cancer [37,39,40]. Increased expression of YKL-40 is reported in advanced tumor stages at both tissue and serum levels in CRC [26], prostate cancer [41,42], and breast cancer [43]. Several studies in the literature support these findings. Ochman et al. linked elevated YKL-40 to immunosuppressive properties within the TME in CRC [44]. Upregulation of YKL-40, but not of YKL-39, is also associated with a metastatic phenotype and poor prognosis in CRC [27,45].

The discrepancy between elevated tissue YKL-39 and reduced plasma levels in CRC is a noteworthy finding. Post-translational modifications, mRNA stability, and protein degradation may all contribute to these findings. The observation of high YKL-40 and low YKL-39 plasma levels suggests differential regulation of these molecules in CRC. The CRC TME is marked by severe inflammation and hypoxia, conditions that strongly promote YKL-40 production [46,47]. In contrast, the TME may suppress YKL-39 gene expression or secretion in its primary source cells [27]. An interesting fact is that YKL-39 is specifically produced by cancer or cancer-associated cells. Tumor-associated macrophages (TAMs) are the main immune source of YKL-40, while M2-type macrophages, which support tumor growth, are significant producers of YKL-39. Kzhyshkowska et al. showed that these macrophages use YKL-39 to stimulate angiogenesis and suppress cytotoxic immune cells [48]. It is highly expressed in the cytoplasm of CRC cells, especially those involved in tumor budding [49], suggesting a role in cancer cell detachment and migration. Cancer-associated fibroblasts (CAFs) and other mesenchymal stromal cells also produce YKL-39, which contributes to the pro-tumor environment by softening the extracellular matrix (ECM) and promoting tumor expansion [46,50]. Therefore, lower plasma levels of YKL-39 may reflect a shift from its role as a circulating inflammatory mediator in healthy tissue to a locally concentrated invasion factor in cancer [51]. Another explanation for the lower plasma YKL-39 levels detected may be the high metabolic and proteolytic activity of the TME. Thus, YKL-39 may undergo rapid turnover, being secreted by M2 macrophages and tumor buds, performing its remodeling function, and then quickly degraded or internalized by neighboring cells through endocytosis [4]. This short-range lifecycle could also result in YKL-39 being consumed at the site of production before it can reach measurable plasma concentrations. In contrast, YKL-40 is induced by various pro-inflammatory cytokines and is secreted either by tumor cells, hepatocytes, neutrophils, and synovial fibroblasts [41,48]. YKL-40 is readily released into the blood, serving as a marker for systemic disease burden and “inflammaging”. It is very soluble and remains stable in circulation, resulting in high serum concentrations. Alternatively, cancer-associated inflammation may induce increased degradation of YKL-39, potentially compensating for elevated YKL-40 production. In fact, YKL-39 lacks a chitinase active site; it is a pseudo-chitinase that binds chitin-oligosaccharides, which may influence its stability. All these findings suggest that despite the structural similarity, YKL-40 and YKL-39 may play opposing roles in cancer progression. Further investigation is required to determine whether reduced YKL-39 levels contribute to the survival of circulating tumor cells, are associated with a more metastatic phenotype, or exert anti-tumoral effects in CRC. These aspects, however, were not the primary focus of the present research.

To date, no previous studies have examined the expression of YKL-39 and YKL-40 in relation to tumor budding alongside their detection in liquid biopsy samples from CRC patients. This investigation offers the first comprehensive mapping of YKL-39 and YKL-40 expression across distinct CRC tumor regions, specifically highlighting their strong enrichment at the tumor front and within tumor buds. This finding is highly significant because tumor budding is a well-established, aggressive feature of CRC, associated with poor prognosis and increased metastatic risk. The CLPs’ spatial localization strongly suggests their potential involvement in EMT and the process of single-cell or small-cluster cell invasion.

Despite the modest cohort size, previously we reported that mesenchymal markers such as N-cadherin and Vimentin, and EMT activators (Twist1, Snai1, and Zeb1) were upregulated in CRC. Based on these results, we hypothesized that YKL-40 expression might be higher in colorectal tumors with more advanced malignancy. On the other side, YKL-39 does not work directly on tumor cells but can influence tumor progression, thus increased YKL-39 levels are associated with increased risk of metastasis and post-chemotherapy drug resistance [18]. In the current survey, the detected association between elevated CLP expressions and tumor budding, coupled with the relationship found between tumor budding and lymphatic invasion, proposes a link between high CLP levels and CRC invasiveness. It raises the hypothesis that these CLPs may act as secreted factors that modify the TME (e.g., via extracellular matrix remodeling) or might directly promote the migratory and invasive phenotype of CRC cells, thereby facilitating access to the lymphatic system, a critical step toward metastasis.

This pilot study is the first to document both gene and protein levels of YKL-39 and YKL-40 in CRC patients’ plasma and WBCs. This direct evidence bridges some gaps between local tumor biology and systemic circulation. Notably, the detection of CLPs in the liquid biopsy samples supports their potential as non-invasive biomarkers. High circulating YKL-40 levels could reflect the total tumor burden, the activity of the invasive front, or the systemic inflammatory response mounted by the WBCs in response to the tumor. Future work should focus on establishing cut-off values and assessing the diagnostic and prognostic accuracy of these plasma/WBCs CLP levels, particularly in relation to the presence or absence of tumor budding in the primary tumor. The lower YKL-39 concentration in the blood reflects the balance between impaired synthesis, secretion, increased breakdown, and clearance. Whether the altered plasma level of YKL-39 is a favorable or unfavorable prognostic marker requires further investigation.

This study also has its limitations. The analysis was restricted by the available data, resulting in a modest sample size that may account for some of the null associations. Despite the sample size constraint, the reliability of the tissue expression data was rigorously maintained. Notably, for the IHC analysis, ten independent visible fields per patient were systematically assessed at 400x magnification, thereby enhancing the internal reliability and representative nature of each patient’s tumor evaluation. Another limitation that warrants consideration is that the study was conducted at a single center, which may affect the generalizability of the findings. There was also limited variability among patients in certain indicators (e.g., G, Bd), which narrowed the scope for modeling. Further independent validation studies utilizing larger, geographically diverse patient cohorts are required to confirm reliability and clarify the ultimate clinical significance of these molecular findings.

In recent years, much research demonstrates that plasma YKL-40 and YKL-39 have significant potential to complement established biomarkers such as CEA (Carcinoembryonic Antigen) and CA 19-9 [52,53]. These molecules may address diagnostic limitations where traditional biomarkers are insufficient, particularly in early-stage negative cases or specific tumor subtypes. Thus, it could be proposed that elevated YKL-40 levels may be considered as an indicator of aggressive tumor biology or recurrence, especially when CEA levels remain within normal ranges; while YKL-39 is an emerging research marker that may facilitate further patient sub-stratification based on immune response profiles. Therefore, CLPs can complement the existing biomarker panel.

Whether YKL-40 and YKL-39 can be integrated into current CRC risk-stratification protocols requires a comprehensive, multi-compartmental approach. YKL-40 functions as a broad systemic indicator, with elevated levels associated with increased systemic inflammatory burden and a higher probability of advanced disease stage or occult metastasis [52]. In contrast, YKL-39 offers a more localized perspective on the mechanical process of tumor invasion. Specifically, a high h-score for YKL-39 in tumor buds identifies aggressive stroma, which may indicate the need for more intensive adjuvant chemotherapy, even in stage II patients [35]. Employing systemic YKL-40 as a diagnostic adjunct and localized YKL-39 as a prognostic marker of invasive potential could substantially enhance the precision of CRC risk stratification. Incorporating these CLPs into established pathological workflows, such as tumor budding assessment, may enable clinicians to more accurately identify high-risk stage II patients who would benefit from intensified adjuvant or systemic therapy. Specifically, the assessment of CLPs can provide a biological snapshot of the tumor’s metastatic potential and can help identify patients at higher risk of invasion or occult nodal spread [54,55].

In summary, this study advances the understanding of the clinical relevance and spatial distribution of YKL-39 and YKL-40 in CRC. Our findings position these CLPs as potential players in the invasive cascade, particularly based on their intensive expression in the tumor front as a surrogate marker for high-grade budding and stromal remodeling, both of which are strongly associated with lymphatic invasion. Beyond their role as mechanistic insights, the detection in liquid biopsy samples provides a compelling rationale for their future validation as accessible, non-invasive diagnostic and prognostic tools. Research must now focus on elucidating the precise molecular pathways by which YKL-39 and YKL-40 facilitate EMT and invasion and on validating their utility in large, prospective patient cohorts, which definitely require further investigation.

## Figures and Tables

**Table 1:** Demographic and clinicopathological characteristics of CRC patients.

Characteristics	*N* (%)/*Mean* ± *SD*
Age (years), mean	71.2 ± 9.8
Sex, *N* (%)	
Female	17 (53.1%)
Male	15 (46.9%)
Primary site, *N* (%)	
Right colon	11 (34.4%)
Left colon	19 (59.4%)
Other (rectum, colon transversum)	2 (6.2%)
Histological grade (G), *N* (%)	
G1–G2 (low)	1 + 29 (93.7%)
G3 (high)	2 (6.3%)
Tumor stage, *N* (%)	
pT1	3 (9.4%)
pT2	1 (3.1%)
pT3	28 (87.5%)
Tumor budding, *N* (%)	
Bd0	1 (5%)
Bd1	1 (5%)
Bd2	8 (40%)
Bd3	10 (50%)

Legend: G1—well-differentiated tumor; G2—moderately differentiated tumor; G3—poorly differentiated tumor.

## Data Availability

All data supporting the findings of the study are available within the paper and Supplementary Files.

## Supplementary Materials

The following supporting information can be downloaded at: https://www.mdpi.com/article/10.3390/cells15030263/s1, Figure S1: Characteristics of CRC patients. (a) Distribution (%) and frequency of CRC patients with tumor grade of differentiation G1—well-differentiated tumor, G2—moderately differentiated tumor and G3—poorly differentiated tumor, in the study group; (b) Distribution (%) and frequency of CRC patients with tumor size pT1, pT2, pT3 and pT4 within the study group; (c) Distribution (%) and frequency of CRC patients with invasion in the lymph nodes pN0, pN1and pN2 within the study group; (d) Distribution (%) and frequency of CRC patients with tumor budding B0, B1, B2 and B3 within the study group; Table S1: Primer sequences used for the qPCR analysis. Integrated DNA Technologies, Leuven, Belgium; Table S2: Uncorrected and Benjamini–Hochberg corrected *p*-values associated with Fisher-Freeman-Halton test of YKL-40 and YKL-39 expression in CRC and normal colon tissues; Table S3: Uncorrected and Benjamini–Hochberg corrected *p*-values associated with Kendall’s tau rank partial correlation between YKL-40 and YKL-39 tissue, plasma, and gene expressions, budding and other clinical variables.
