# Supplementary material for: Chitinase-like Proteins YKL-40 and YKL-39 in Colorectal Cancer
Source: Cells. 2026 Jan 30;15(3):263. doi: 10.3390/cells15030263 (PMC12971110; doi:10.3390/cells15030263)
Supplement: Supplementary file 1 [file cells-15-00263-s001.zip › Supplementary Table S1.pdf]

Supplementary Table S1.

Primer sequences used for the qPCR analysis. Integrated DNA Technologies, Leuven, Belgium

No	GENE NAME	PRIMER SEQUENCE 5`- 3`	AMPLICON SIZE (bp)	EFFICIENCY (%)	CORRELATION COEFICIENT (R^2)
1	CHI3L1 Fw	5`- CTGCTCCAGTGCTGCTCT - 3`	163	91.3	0.969
2	CHI3L1 Rev	5`- TACAGAGGAAGCGGTCAAGG - 3`			
3	CHI3L2 Fw	5′- CCCATCACAGAGTCTTCAGG -3′	181	93.1	1.000
4	CHI3L2 Rev	5′- GGATCAGCAGGTTCCCTACG -3′			
5	GAPDH Fw	5`-AGGTCCACCACTGACACGTTG-3	125	92.0	0.962
6	GAPDH Rev	5`- AGCTGAACGGGAAGCTCACT - 3`			
7	ACTINB Fw	5`- AGTGTGACG TGGACATCCGGA - 3`	211	107.2	0.952
8	ACTINB Rev	5`- GCCAGGGCAGTGATCTCCTCCT - 3`			
9	hUBC Fw	5`- TCCTGATCAGGCAGAGGTTGATCTT - 3`	188	90.1	0.999
10	hUBC Rev	5`- GGACCAAGTGCAGAGTGGACTCTT - 3`			
