# Supplementary material for: Chitinase-like Proteins YKL-40 and YKL-39 in Colorectal Cancer
Source: Cells. 2026 Jan 30;15(3):263. doi: 10.3390/cells15030263 (PMC12971110; doi:10.3390/cells15030263)
Supplement: Supplementary file 1 [file cells-15-00263-s001.zip › Supplementary Table S2.pdf]

Supplementary Table S2

Uncorrected and Benjamini–Hochberg corrected p-values associated with *Fisher-Freeman-Halton test* of YKL-40 and YKL-39 expression in CRC and normal colon tissues.

↓ labels (optional) ↓	↓ P-values ↓	Benjamini-Hochberg significance	Benjamini-Hochberg P-value
YKL40 TP v YKL-40TS	0.0001	significant	0.0003
YKL40 TS v YKL-40 TF	0.0001	significant	0.0003
YKL40 TP v YKL-40 TF	0.0001	significant	0.0003
YKL40 TP v YKL-40 TNC	0.0001	significant	0.0003
YKL40 TF v YKL-40 TNC	0.0001	significant	0.0003
YKL39 TP v YKL39 TF	0.0001	significant	0.0003
YKL39 TS v YKL39 TF	0.0001	significant	0.0003
YK39 TP v YKL39 TNC	0.0001	significant	0.0003
YKL39 TS v YKL39 TNC	0.0001	significant	0.0003
YKL39 TS v YKL39 TNC	0.0001	significant	0.0003
YKL39 TF v YKL39 TNC	0.0001	significant	0.0003
YKL40 TS v YKL-40 TNC	0.0002	significant	0.00048
YK39 TP v YKL39 TS	0.0002	significant	0.00048
