# Supplementary material for: Chitinase-like Proteins YKL-40 and YKL-39 in Colorectal Cancer
Source: Cells. 2026 Jan 30;15(3):263. doi: 10.3390/cells15030263 (PMC12971110; doi:10.3390/cells15030263)
Supplement: Supplementary file 1 [file cells-15-00263-s001.zip › Supplementary Table S3.pdf]

Supplementary Table S3

Uncorrected and Benjamini–Hochberg corrected p-values associated with Kendall's tau rank partial correlation between YKL-40 and YKL-39 tissue, plasma, and gene expressions, budding and other clinical variables

Var1	Var2	Tau	P_value	CI_lower	CI_upper	BH_adjusted
Budd	G	-0,267	0,134			0,378
Budd	pN	0,351	0,058	-0,107	0,640	0,325
Budd	pT	0,179	0,317	**0,008**	**0,369**	0,582
Budd	YKL_40_mRNA	0,097	0,600	-0,368	0,503	0,780
Budd	YKL39_ELISA	0,087	0,650	-0,362	0,445	0,806
Budd	YKL39_mRNA	0,057	0,750	-0,391	0,446	0,855
Budd	YKL39_TF	0,548	**0,002**	-0,012	0,796	**0,048**
Budd	YKL39_TNC	0,197	0,269	-0,321	0,578	0,537
Budd	YKL39_TP	0,081	0,650	-0,382	0,377	0,806
Budd	YKL39_TS	0,180	0,313	-0,370	0,580	0,582
Budd	YKL40_ELISA	0,060	0,735	-0,409	0,333	0,855
Budd	YKL40_TF	0,111	0,534	-0,308	0,508	0,751
Budd	YKL40_TNC	-0,363	**0,042**	-0,664	0,065	0,293
Budd	YKL40_TP	0,286	0,109	-0,145	0,644	0,356
Budd	YKL40_TS	0,367	**0,040**	-0,188	0,681	0,293
G	pN	0,203	0,225	**0,072**	**0,213**	0,475
G	pT	0,676	**0,000**	**0,239**	**0,786**	**0,003**
G	YKL_40_mRNA	-0,036	0,827	-0,232	0,064	0,891
G	YKL39_ELISA	-0,143	0,408	**-0,332**	**-0,052**	0,682
G	YKL39_mRNA	0,201	0,215	0,000	0,297	0,467
G	YKL39_TF	NA	NA			NA
G	YKL39_TNC	NA	NA			NA
G	YKL39_TP	NA	NA			NA
G	YKL39_TS	NA	NA			NA
G	YKL40_ELISA	-0,200	0,217	-0,252	0,136	0,467
G	YKL40_TF	NA	NA			NA
G	YKL40_TNC	NA	NA			NA
G	YKL40_TP	NA	NA			NA
G	YKL40_TS	NA	NA			NA
pN	pT	0,210	0,208	**0,105**	**0,365**	0,466
pN	YKL_40_mRNA	0,054	0,755	-0,347	0,389	0,855

pN	YKL39_ELISA	0,156	0,382	-0,298	0,499	0,651
pN	YKL39_mRNA	-0,086	0,606	-0,441	0,359	0,780
pN	YKL39_TF	0,215	0,245	-0,290	0,602	0,498
pN	YKL39_TNC	0,197	0,287	-0,253	0,629	0,565
pN	YKL39_TP	0,027	0,883	-0,525	0,316	0,916
pN	YKL39_TS	0,100	0,591	-0,444	0,517	0,780
pN	YKL40_ELISA	-0,030	0,857	-0,413	0,416	0,914
pN	YKL40_TF	-0,015	0,937	-0,479	0,422	0,954
pN	YKL40_TNC	-0,111	0,549	-0,498	0,302	0,760
pN	YKL40_TP	0,239	0,197	-0,224	0,614	0,461
pN	YKL40_TS	0,098	0,596	-0,405	0,421	0,780
pT	YKL_40_mRNA	-0,036	0,827	-0,232	0,064	0,891
pT	YKL39_ELISA	-0,267	0,122	**-0,473**	**-0,052**	0,378
pT	YKL39_mRNA	0,237	0,144	**0,069**	**0,429**	0,378
pT	YKL39_TF	0,289	0,106	**0,141**	**0,402**	0,356
pT	YKL39_TNC	-0,291	0,103	**-0,505**	**-0,156**	0,356
pT	YKL39_TP	0,076	0,668	**-0,227**	**-0,056**	0,814
pT	YKL39_TS	-0,111	0,533	-0,228	0,101	0,751
pT	YKL40_ELISA	-0,293	0,071	**-0,529**	**-0,104**	0,340
pT	YKL40_TF	-0,318	0,075	**-0,382**	**-0,135**	0,340
pT	YKL40_TNC	-0,289	0,106	**-0,521**	**-0,239**	0,356
pT	YKL40_TP	0,156	0,384	**0,117**	**0,345**	0,651
pT	YKL40_TS	0,355	**0,047**	**0,201**	**0,456**	0,307
YKL_40_mRNA	YKL39_ELISA	0,228	0,202	**0,089**	**0,584**	0,461
YKL_40_mRNA	YKL39_mRNA	-0,044	0,795	-0,393	0,363	0,881
YKL_40_mRNA	YKL39_TF	-0,218	0,239	-0,494	0,478	0,496
YKL_40_mRNA	YKL39_TNC	-0,319	0,085	-0,561	0,178	0,352
YKL_40_mRNA	YKL39_TP	-0,002	0,990	-0,561	0,128	0,990
YKL_40_mRNA	YKL39_TS	-0,272	0,142	-0,477	0,317	0,378
YKL_40_mRNA	YKL40_ELISA	0,320	0,056	**0,141**	**0,627**	0,325
YKL_40_mRNA	YKL40_TF	-0,295	0,111	-0,535	0,390	0,356
YKL_40_mRNA	YKL40_TNC	-0,247	0,183	-0,584	0,192	0,445
YKL_40_mRNA	YKL40_TP	-0,266	0,150	-0,485	0,246	0,383
YKL_40_mRNA	YKL40_TS	-0,360	0,052	-0,549	0,340	0,323
YKL39_ELISA	YKL39_mRNA	0,016	0,925	-0,308	0,318	0,951
YKL39_ELISA	YKL39_TF	0,110	0,567	-0,255	0,556	0,775
YKL39_ELISA	YKL39_TNC	-0,030	0,875	-0,436	0,420	0,916

YKL39_ELISA	YKL39_TP	0,424	**0,028**	-0,033	0,316	0,245
YKL39_ELISA	YKL39_TS	0,048	0,804	-0,237	0,415	0,883
YKL39_ELISA	YKL40_ELISA	0,420	**0,015**	-0,029	0,702	0,196
YKL39_ELISA	YKL40_TF	0,149	0,438	-0,260	0,606	0,710
YKL39_ELISA	YKL40_TNC	0,186	0,333	-0,236	0,425	0,602
YKL39_ELISA	YKL40_TP	-0,281	0,145	-0,541	0,237	0,378
YKL39_ELISA	YKL40_TS	-0,181	0,347	-0,453	0,312	0,608
YKL39_mRNA	YKL39_TF	-0,127	0,475	-0,463	0,300	0,715
YKL39_mRNA	YKL39_TNC	-0,261	0,144	-0,618	0,238	0,378
YKL39_mRNA	YKL39_TP	0,431	**0,016**	**0,184**	**0,620**	0,196
YKL39_mRNA	YKL39_TS	-0,456	**0,011**	**-0,723**	**-0,018**	0,170
YKL39_mRNA	YKL40_ELISA	-0,152	0,348	-0,470	0,234	0,608
YKL39_mRNA	YKL40_TF	-0,386	**0,031**	-0,651	0,092	0,245
YKL39_mRNA	YKL40_TNC	-0,047	0,791	-0,426	0,385	0,881
YKL39_mRNA	YKL40_TP	-0,317	0,076	-0,647	0,157	0,340
YKL39_mRNA	YKL40_TS	-0,120	0,501	-0,487	0,357	0,728
YKL39_TF	YKL39_TNC	0,304	0,089	-0,180	0,679	0,355
YKL39_TF	YKL39_TP	0,131	0,463	-0,362	0,456	0,715
YKL39_TF	YKL39_TS	0,660	**0,000**	**0,392**	**0,870**	**0,012**
YKL39_TF	YKL40_ELISA	-0,098	0,582	-0,449	0,396	0,780
YKL39_TF	YKL40_TF	0,080	0,655	-0,305	0,545	0,806
YKL39_TF	YKL40_TNC	-0,250	0,161	-0,629	0,275	0,400
YKL39_TF	YKL40_TP	0,188	0,293	-0,344	0,569	0,567
YKL39_TF	YKL40_TS	0,532	**0,003**	-0,029	0,809	0,054
YKL39_TNC	YKL39_TP	-0,057	0,751	-0,495	0,319	0,855
YKL39_TNC	YKL39_TS	0,582	**0,001**	**0,369**	**0,741**	**0,031**
YKL39_TNC	YKL40_ELISA	-0,128	0,475	-0,497	0,368	0,715
YKL39_TNC	YKL40_TF	0,134	0,453	-0,349	0,543	0,715
YKL39_TNC	YKL40_TNC	0,126	0,481	-0,351	0,511	0,715
YKL39_TNC	YKL40_TP	0,297	0,096	-0,231	0,625	0,356
YKL39_TNC	YKL40_TS	0,125	0,485	-0,445	0,462	0,715
YKL39_TP	YKL39_TS	-0,326	0,067	**-0,664**	**-0,023**	0,340
YKL39_TP	YKL40_ELISA	-0,146	0,414	-0,541	0,355	0,682
YKL39_TP	YKL40_TF	-0,289	0,105	**-0,608**	**-0,208**	0,356
YKL39_TP	YKL40_TNC	0,072	0,685	-0,137	0,518	0,817
YKL39_TP	YKL40_TP	-0,406	**0,023**	**-0,770**	**-0,248**	0,245
YKL39_TP	YKL40_TS	-0,182	0,309	**-0,510**	**-0,122**	0,582
YKL39_TS	YKL40_ELISA	-0,027	0,878	-0,446	0,421	0,916
YKL39_TS	YKL40_TF	0,268	0,133	-0,204	0,607	0,378
YKL39_TS	YKL40_TNC	-0,110	0,536	-0,586	0,352	0,751

YKL39_TS	YKL40_TP	0,314	0,079	-0,238	0,681	0,340
YKL39_TS	YKL40_TS	0,387	**0,030**	-0,576	0,808	0,245
YKL40_ELISA	YKL40_TF	0,072	0,686	-0,257	0,477	0,817
YKL40_ELISA	YKL40_TNC	0,128	0,473	-0,254	0,464	0,715
YKL40_ELISA	YKL40_TP	0,088	0,623	-0,374	0,429	0,793
YKL40_ELISA	YKL40_TS	0,005	0,977	-0,318	0,509	0,986
YKL40_TF	YKL40_TNC	0,228	0,202	-0,360	0,589	0,461
YKL40_TF	YKL40_TP	0,387	**0,030**	-0,085	0,724	0,245
YKL40_TF	YKL40_TS	0,326	0,067	-0,102	0,701	0,340
YKL40_TNC	YKL40_TP	0,271	0,129	-0,384	0,610	0,378
YKL40_TNC	YKL40_TS	-0,063	0,724	-0,583	0,391	0,854
YKL40_TP	YKL40_TS	0,636	**0,000**	**0,351**	**0,849**	**0,014**
