# Supplementary figures and images for: Chitinase-like Proteins YKL-40 and YKL-39 in Colorectal Cancer
Source: Cells. 2026 Jan 30;15(3):263. doi: 10.3390/cells15030263 (PMC12971110; doi:10.3390/cells15030263)

## Slide 1
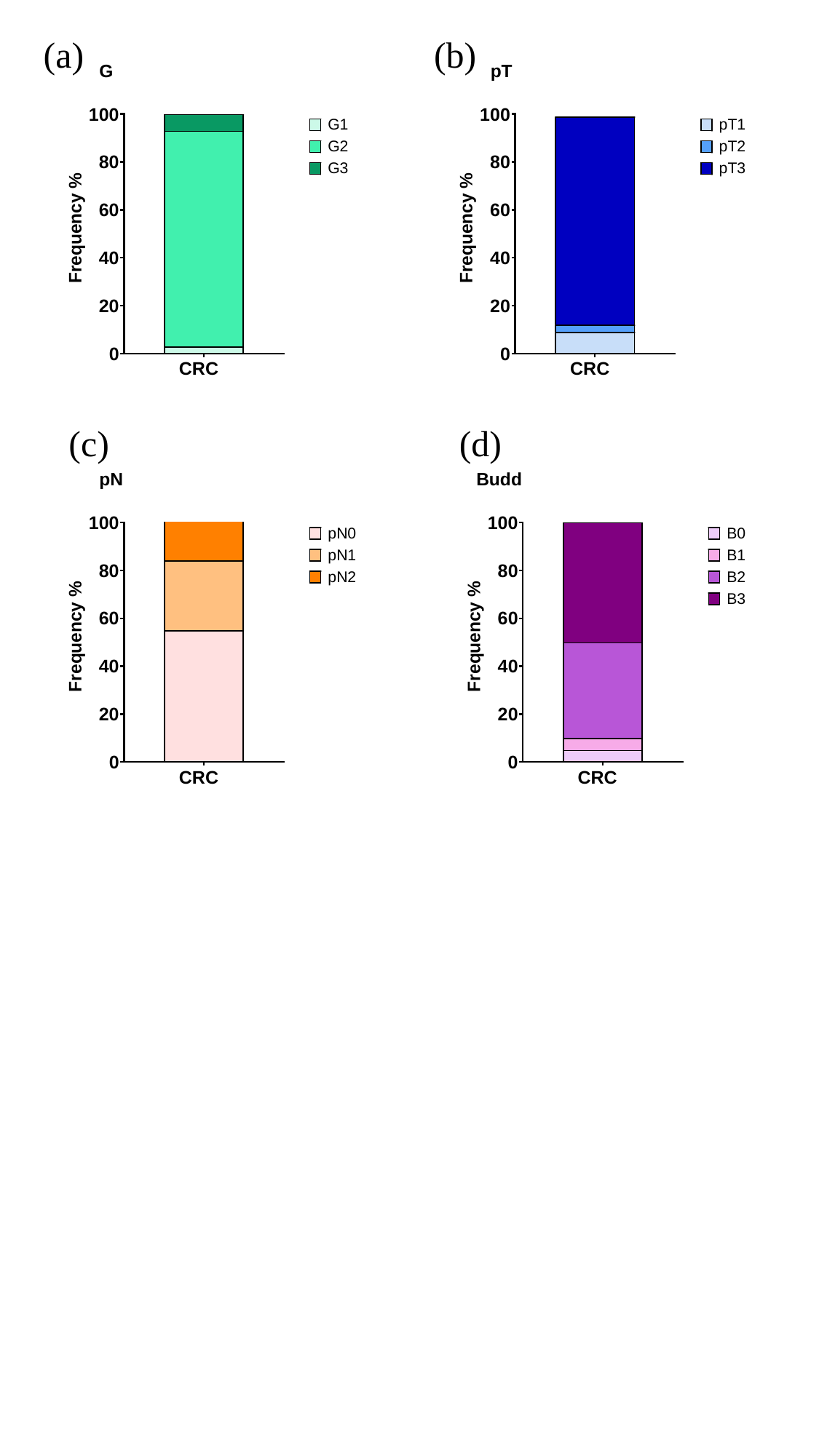

(a)
(b)
(c)
(d)

Supplement: Supplementary file 1 [file cells-15-00263-s001.zip › Supplementary Figure S1.pptx]
